# The effect of neoadjuvant platinum-based chemotherapy in *BRCA* mutated triple negative breast cancers -systematic review and meta-analysis

**DOI:** 10.1186/s13053-019-0111-y

**Published:** 2019-03-25

**Authors:** Olga Caramelo, Cristina Silva, Francisco Caramelo, Cristina Frutuoso, Teresa Almeida-Santos

**Affiliations:** 1 Gynecology Department, Coimbra Hospital and University Centre (CHUC), EPE, Praceta Prof. Mota Pinto, 3000-075 Coimbra, Portugal; 2 Faculty of Pharmacy of the University of Coimbra, Rua Filipe Simões n° 33, 3000-186 Coimbra, Portugal; 3 Laboratory of Biostatistics and Medical Informatics, iCBR – Faculty of Medicine, University of Coimbra, 3000-354 Coimbra, Portugal; 4 Centre for Fertility Preservation, Human Reproduction Department, Coimbra Hospital and University Centre (CHUC), EPE, Praceta Prof. Mota Pinto, 3000-075 Coimbra, Portugal; 5 Faculty of Medicine of the University of Coimbra, Azinhaga de Santa Comba - Celas, 3000-548 Coimbra, Portugal

**Keywords:** Triple negative breast cancer, *BRCA*, Neoadjuvant chemotherapy, Cisplatin, Carboplatin

## Abstract

**Background:**

Triple negative breast cancers (TNBC) are associated with an aggressive clinical course, earlier recurrence and short survival. *BRCA* – mutated tumours represent up to 25% of all TNBC. *BRCA* status is being studied as a predictive biomarker of response to platinum agents. However, the predictive role of *BRCA* status is still uncertain in this setting. Since TNBC is a very heterogeneous group of diseases, it is important to identify subsets of TNBC patients that may benefit from platinum-based therapy. This study aims to establish if the presence of a germline *BRCA* mutation in women with TNBC improves the pathologic complete response (pCR) after neoadjuvant chemotherapy with platinum compounds.

**Methods:**

An extensive literature search was performed in MEDLINE, EMBASE and LILACS databases, WHO (WHO International Clinical Trials Registry Platform) and the Cochrane Controlled Trials Register Database, for online trial registries and conference proceedings. The measurement of pCR was assessed by pathology review of breast specimen and lymph nodes.

**Results:**

The overall OR was computed using random effects models.

Seven studies were included, comprising a total of 808 TNBC patients, among which 159 were *BRCA* mutated. Among mutated TNBC patients, 93 (93/159; 58.4%) achieved pCR, while 410 wildtype patients (410/808; 50.7%) showed pCR (OR 1.459 CI 95% [0.953–2.34] *p* = 0.082) although this result did not reach statistical significance.

**Conclusions:**

This meta-analysis shows that the addition of platinum to chemotherapy regimens in the neoadjuvant setting increases pCR rate in *BRCA –* mutated as compared to wild-type TNBC patients. However, this trend did not achieve statistical significance.

**Trial registration:**

CRD42018092341

## Background

Triple-negative breast cancer (TNBC) is a subtype of breast cancer that accounts for approximately 15 to 20% of all breast cancers [1, 2]. TNBC is defined as lacking the expression of both estrogen receptor (ER), progesterone receptor (PR), and human epidermal growth factor receptor 2 (HER2). This type of breast tumours comprises a matter of clinical interest because no effective targeted therapy is available, being therefore associated with a more aggressive clinical course, earlier recurrence and short survival, when compared with other types of breast cancer [3, 4]. TNBC has close associations with specific subgroups of patients, including premenopausal women and *BRCA* mutation carriers [5, 6].

The standard treatment for TNBC that has been shown to improve outcomes is chemotherapy. Interestingly, despite the poor prognosis, it is evident that a subset of patients appears to be particularly chemosensitive when compared to ER positive breast cancers [7]. Hence neoadjuvant chemotherapy has become a standard approach to treat patients with TNBC. It is worthy of note that patients who achieve pathologic complete response (pCR) after neoadjuvant treatment have been shown to have improved long-term outcomes when compared to patients with residual invasive disease [8, 9].

The usual combinations of chemotherapy in the treatment of TNBC include anthracycline and taxane-based regimens. Adding platinum derivatives in the neoadjuvant setting has been shown to improve pCR and also disease-free survival (DFS) in TNBC [10, 11]. Despite these promising results, the inclusion of carboplatin as part of neoadjuvant therapy regimens has not been consensually accepted.

There is evidence that TNBC comprises a heterogeneous breast cancer subtype and this heterogeneity may partially explain the lack of success of targeted therapies in unselected patients [12, 13]. TNBC presents as multiple clinically, biologically and molecular subtypes, that are not yet clearly defined and that may differently respond to chemotherapy. Some patients respond very well to neoadjuvant chemotherapy, with high pCR after surgery. Nevertheless, other patients show no response to neoadjuvant treatment and suffer from early relapse [2].

Disappointingly, a predictive factor for patients who will better respond to specific chemotherapy has not been identified. Further research on the heterogeneity within TNBC may lead to different therapeutic strategies. Indeed, 75 to 80% of TNBC belong to the basal-like subtype of breast cancer, which includes specific genomic profiles, being the most frequent mutations in the *BRCA1*, *TP53* and *PIK3CA* genes [13, 14].

Approximately 60 to 80% of breast cancer patients with *BRCA*1 germline mutations have triple negative cancers [15, 16]. On the other hand, around 15 to 25% of all TNBC patients harbour a *BRCA* mutation [17–19].

A higher pCR rate was demonstrated in TNBC after neoadjuvant chemotherapy, when compared with others subtypes of breast cancer [8]. Moreover, pCR is considered a predictive factor for future outcomes: patients who achieve pCR with neoadjuvant chemotherapy have significant better overall survival than those with residual disease [9]. These observations suggest pCR to be a prognostic factor for breast cancer and that subgroup of TNBC patients more sensitive to chemotherapy possibly exists.

Recent evidence suggests that TNBC and *BRCA* 1 related DNA- repair defects determine sensitivity to DNA –damaging agents, such as platinum- based chemotherapy and poly (ADP ribose) polymerase (PARP) inhibitors [10, 20, 21]. *BRCA* mutations are responsible for a defect in DNA double-strand break repair which seems to make this type of breast cancer particularly sensitive to treatment with inter-strand cross-linking agents, including platinum analogues. Despite this apparent sensitivity to neoadjuvant chemotherapy, the exact role of carboplatin is still under debate [22]. The incorporation of platinum compounds increases pCR rates in TNBC and *BRCA* mutated patients; however, in neoadjuvant setting its use remains controversial and it is not routinely recommended in unselected TNBC or *BRCA* mutations carriers [23–25].

Since TNBC is a very heterogeneous group of diseases, it is urgent to identify subsets of TNBC patients that may benefit from platinum-based therapy and their eventual predictors of response. With this purpose, we have conducted a systematic review of studies including TNBC patients with germline *BRCA* mutation, in order to determine predictive factors for platinum based therapy response.

Our primary aim was to establish if the presence of a germline *BRCA* mutation in women with TNBC does improve the pCR after neoadjuvant chemotherapy with platinum compounds. Additionally, we aimed to recognize if the use of platinum compounds could help predict pCR in a subgroup of breast cancer patients in a neoadjuvant setting.

There are several studies and meta-analysis that report the association between neoadjuvant chemotherapy and pCR for TNBC, but until now none has been designed with the specific purpose of comparing pCR in TNBC wild-type and TNBC *BRCA* mutated tumours, after exposure to the same neoadjuvant platinum-based regimen.

Although numerous clinical studies have reported the superior efficacy of platinum- based treatment for TNBC, most of the results were observed in small samples. Here we present a systematic review with meta-analysis of studies that include a subset of TNBC patients with *BRCA* mutations.

## Material and methods

We have conducted a systematic literature review following both Cochrane and PRISMA-P guidelines [26].

### Protocol and registration

Two reviewers (OC and CS) established the protocol for this systematic review, which was published at the International Prospective Register of Systematic Reviews (PROSPERO) in April 2018 with the registration number CRD42018092341.

### Eligibility criteria

The following eligibility criteria were defined according to the PICO methodology:

- Patients: women with non-metastatic TNBC (stage I-III) who have been submitted to neoadjuvant chemotherapy with platinum regimens (including cisplatin or carboplatin) and were previously tested for the *BRCA*1 mutation.
- Outcomes: studies must report data on pCR, as determined by histological study after surgery; pCR was defined as the absence of any residual invasive cancer (ypT0/is ypN0) or of any invasive and non-invasive cancer (ypT0 ypN0), on evaluation of the resected breast specimen and of all sampled ipsilateral lymph nodes following completion of neoadjuvant therapy. pCR was used as the indicator of response rate among TNBC with or without *BRCA* 1 mutation when treated with platinum compounds in a neoadjuvant chemotherapy setting. When described, Residual Cancer Burden index (RCB) was used as a secondary outcome. This index evaluates the extent of residual disease and has been validated as an independent prognostic marker of distant relapse- free survival in patients with breast cancer treated with neoadjuvant chemotherapy (RCB-0 complete pathologic response; RCB-I minimal residual disease; RCB II moderate residual disease; RCB III extensive residual disease). Only RCB 0 and RCB I were included as criteria of response.
- Studies: we have included phase II and III clinical trials, retrospective studies and cohort studies as long as they include comparison of TNBC *BRCA* 1 mutated vs TNBC non-mutated subgroups. Single arm trials or case reports were not included. Only studies in English language were considered, published in a time frame of 10 years (2008–2018).

### Search strategy and study selection

An extensive bibliographic search was performed in the Medline, EMBASE and LILACS databases, and in WHO (WHO International Clinical Trials Registry Platform) and Cochrane (Cochrane Controlled Trials Register Database). The following Medical Subject Heading terms were used: “breast neoplasm”, “breast cancer”, “breast carcinoma”, “breast tumor” or “breast tumour”, combined with “triple negative” or “TNBC” and “platinum compounds”, “cisplatin”, “carboplatin”, or “platinum”. Furthermore, a search for relevant abstracts was performed in the conference proceedings from the European Society for Medical Oncology (ESMO) congress, the American Society of Clinical Oncology (ASCO) annual meeting, and the San Antonio Breast Cancer Symposium (SABCS).

### Data extraction

Two researchers (OC and CS) performed the search independently and any discrepancies during the process were resolved by discussion with a third reviewer (TAS).

**Fig. 1:**
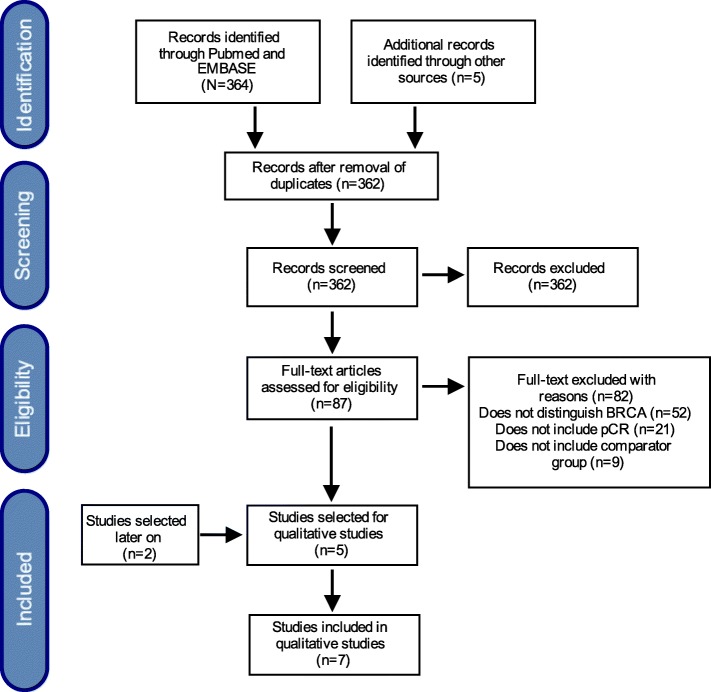
Flowchart of the literature search

Studies were screened for inclusion over three phases, using EndNote® software: 1) duplicates were searched and deleted; 2) the two reviewers OC and CS screened the results first by title and then by abstract; when a title seemed relevant, the abstract was reviewed for eligibility; 3) if any doubt remained, the full text of the article was retrieved and discussed. Arbitration by a third author (TAS) was applied in case of persistent disagreement. The reasons for exclusion were recorded and present in (Fig. 1).

**Fig. 2:**
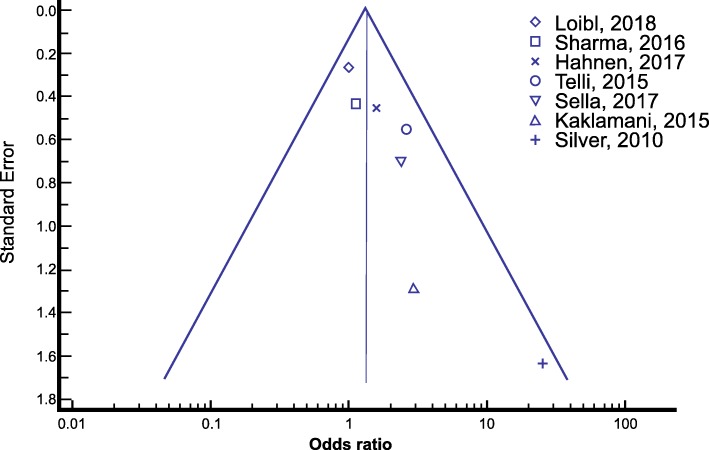
Funnel plot of publication bias for Odds Ratio meta-analysis

Data extraction was performed between April 2017 and June 2018. The following information was independently extracted from each article: 1) basic information, including year of publication and first author’s name; 2) study information, including setting, sample size, study design, inclusion and exclusion criteria, number of TNBC wild-type patients and number of TNBC patients with *BRCA* mutation; 3) treatment information, including neoadjuvant platinum-based regimens and number of cycles, type of surgery; 4) outcomes of interest: number of patients achieving pCR in the TNBC wildtype and *BRCA* mutated subgroups; number of patients with RCB 0 or 1in the same subgroups. A funnel-plot was performed to assess the potential publication bias (Fig. 2).

### Statistical analysis

The primary outcome measure was pCR assessed by pathologic review of breast specimen and lymph nodes. Study-specific Odds Ratio (OR) of pCR/RCB was estimated indirectly from the total number of patients and the number of patients with pCR/RCB 0 or 1 in each arm (wildtype TNBC/*BRCA* mutated TNBC).

The overall OR was reported with a measure of precision (confidence interval or standard error). Statistical analysis was performed using the software R®, version 3.5.0. The software Medcalc was also used for forest- plots and funnel plot generation. Meta-analysis was conducted using a random effects model. Statistical heterogeneity was assessed with a chi-square test. The significance level adopted was 0.05.

## Results

A total of 362 references were identified through database search. Eighty-seven articles were assessed for eligibility, among them 82 were excluded, mainly because they did not report pCR or did not specifically discriminate the presence of *BRCA* mutation in TNBC. Among these, five studies were selected for the review and two new publications were added later on, during the execution of this systematic review (Table 1).

**Table 1:** Characteristics of the studies included in this work

	Affiliation	Type of study	Stage of disease	Median age	TNBC patients (control arm)	TNBC patients with *BRCA 1/2* mutations (experimental arm)	Endpoints available for inclusion	Platinum
Silver [44]	USA	Clinical Trial	II-III	50	26	2	pCR	cisplatin
Telli [42]/PrECOG 0105	USA	Ph. II Clinical Trial	I-IIIA	48	73	17	pCR, RCB	carboplatin
Kaklamani [41]/NCT01372579	USA	Ph. II Clinical Trial	I-III	52,5	27	3	pCR, RCB	carboplatin
Sharma [45]/PROGECT	USA, Spain	Clinical trial	I-III	51	133	27	pCR, RCB	carboplatin
Hahnen [21]/Gepar Sixto	Germany	Ph. II RCT	II-III	48	120	26	pCR, DFS	carboplatin
Loibl [28]/BrighTNess	15 countries North America Europe Asia-Pacific	Ph. III RCT	II-III	segment I 51 segment II 49	406	70	pCR, EFS, OS	carboplatin
Sella [46]	Israel	Clinical trial	I-III	42.3	23	14	pCR	carboplatin

One study [27] that assessed pCR in TNBC *BRCA* mutated patients only included one patient submitted to neoadjuvant chemotherapy with cisplatin and was excluded from the study.

### Studies and outcomes

Most of the studies only presented pCR rate and/or RCB as efficacy outcomes. In the study by Hannen et al. [21], disease free survival (DFS) was also reported. Loibl and colleagues [28] indicated that results on event –free survival (EFS) and overall survival (OS) were ongoing.

### Pathologic complete response (pCR) rate

Overall, seven studies were included, comprising a total of 808 TNBC patients, among which 159 were *BRCA* mutated (Table 2).

**Table 2:** Detailed characteristics of studies included regarding treatment regimens, efficacy (pCR) outcomes and safety outcomes in *BRCA-*mutated and wild-type TNBC groups

Author / Year	Chemotherapy regimen- drug and dose	pCR wild-type TNBC (control group)	pCR *BRCA* 1/2 TNBC mutation	Adverse events
Silver [44]	Four cycles of Cisplatin at 75 mg/m^2^ every 21 days	4/26–15.4%	2/2–100%	Severe toxicity was uncommon. One patient had a grade 4 elevation of AST/AST. There were nine grade 3 toxicities reported: tinnitus, neutropenia, fatigue, hyperkalemia, elevation of ALT/ALT, nausea, myalgia, skin toxicity, and GI toxicity.
Telli [42]/ PrECOG 0105	Four cycles of carboplatin (on days 1 and 8) + gemcitabin (1000 mg/m2 on days 1 and 8), + iniparib (5.6 mg/Kg on days 1,4,8, and 11) every 21 days	22/73–33%	9/17–53%	All patients had at least one treatment-emergent adverse event (TEAE). The most common treatment-related TEAEs were fatigue, nausea, neutropenia or neutrophil count decreased, alopecia, anemia, dysgeusia, diarrhea and rash. All grade 4 TEAEs occurred in patients receiving six cycles of treatment. There were no deaths during the study.
Kaklamani [41]/NCT01372579)	Four cycles of carboplatin AUC 6 iv + eribulin 1.4 mg/m^2^ (day 1 and 8) every 21 days	11/27–40%	2/3–66.7%	Overall the combination was well tolerated with mostly grade 1 and 2 toxicities. One patient had febrile neutropenia. Overall eribulin was dose reduced in 13 patients and carboplatin was dose reduced in eight patients mostly due to neutropenia. One patient received only one cycle of therapy and discontinued due to intolerance.
Sharma [45]/ PROGECT	Six cycles of Carboplatin AUC 6 + docetaxel 75 mg/m^2^ every 21 days	75/ 133–56.3%	16/27–59.3%	Twenty-eight percent of patients experienced one or more grade 3/4 adverse events. Eighty-three percent of patients completed all 6 cycles of treatment. Twelve percent of patients discontinued treatment prematurely (6.0% because of toxicity). No treatment related deaths were reported
Hahnen [21]/ Gepar Sixto	Carboplatin AUC 1–5 + paclitaxel 80 mg/m^2^+ doxorubicin 20 mg/m^2^once a week for 18 weeks+ bevacizumab 15 mg/kg iv every 3 weeks	66/120–55%	17/26–65.4%	Grade 3 or 4 neutropenia, grade 3 or 4 anaemia, grade 3 or 4 thrombocytopenia and grade 3 or 4 diarrhoea were significantly more common in the carboplatin group than in the no-carboplatin group.
Loibl [28]/ BrighTNess	Segment I-Paclitaxel 80 mg/m2 weekly for 12 doses+ Carboplatin AUC 6 every 3 weeks for four cycles+ veraparib 50 mg orally twice a day. Segment II-Paclitaxel 80 mg/m2 weekly for 12 doses+ Carboplatin AUC 6 every 3 weeks for four cycles	142/270 (52.3%) 80/136 (58.8%) (Total 222/406–54.7%)	26/46–56.5% 12/24–50% (Total 38/70–54.3%)	Incidences of haematological and gastrointestinal adverse events (neutropenia, thrombocytopenia, anaemia, nausea, and vomiting) were increased with carboplatin containing regimens during segment 1 treatment.
Sella [46]	Four cycles of doxorubicin (60 mg/m2) and cyclophosphamide (600 mg/m2) every 2 weeks 12 weekly cycles of paclitaxel (80/m2) with carboplatin (AUC 1.5)	10/23 (45.0%)	10/14 (64.0%)	Grade 3/4 neutropenia was the most common haematological toxicity and observed in 42.5% of patients, however only two cases were complicated with fever. No treatment-related deaths were reported.

*AUC* Area Under the Curve, *pCR* Pathologic complete response

**Fig. 3:**
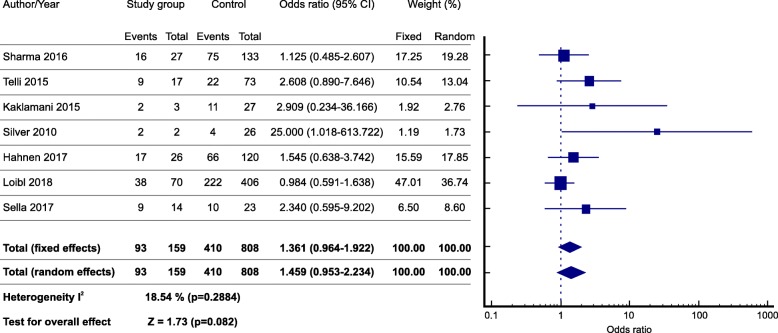
Forest plot of odds ratio for pathologic complete response of *BRCA –* mutated and *BRCA* wild-type TNBC after neoadjuvant platinum- based chemotherapy

Among mutated TNBC patients, 93 (93/159;58.4%) achieved pCR, whereas 410 wildtype patients (410/808; 50.7%) showed pCR (OR = 1.459 CI [95% 0.953–2.34] *p* = 0.082) although this result did not reach statistical significance (Fig. 3).

No significant heterogeneity was found between studies (*I*^*2*^ = 18.54%; *p* = 0.2884). The symmetric funnel plot for this meta-analysis is an additional indicator of the absence of publication bias and study heterogeneity [29].

### Residual cancer burden (RCB) rate

**Fig. 4:**
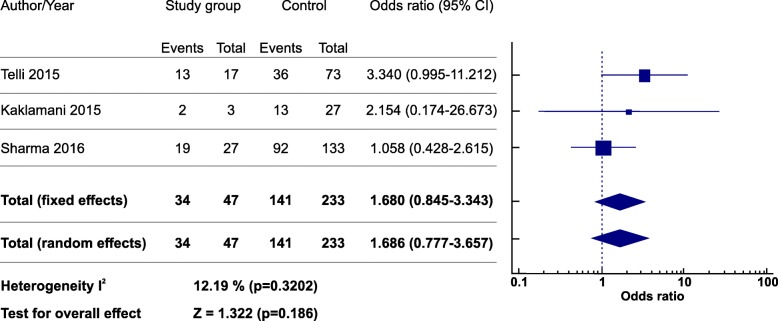
Forest plot of odds ratio for residual cancer burden of *BRCA –* mutated and *BRCA* wild-type TNBC after neoadjuvant platinum-based chemotherapy

Three of the included studies evaluated RCB as an outcome. From a total of 233 TNBC patients in which RCB was evaluated, 47 patients were *BRCA*-mutated. In mutated TNBC patients, 34 (34/47; 72.3%) achieved RCB 0/1, while 141(141/233; 60.5%) wild type TNBC attained RCB. Figure 4 shows an OR 1.68 (CI 95% [0.777–3.657], *p* = 0.186) favoring the use of platinum-based therapy in the mutated TNBC group, although this result was not statistically significant. No significant heterogeneity among studies was observed (*I*^*2*^ = 12.19%; *p* = 0.320).

### Safety outcomes

An overview of the most common adverse events after treatment with the different platinum-based chemotherapy regimens is presented in Table 2. The *BRCA* status of patients experiencing adverse events was not identified in any of the included studies.

## Discussion

In this systematic review with meta-analysis, we intended to clarify the role of platinum- based chemotherapy as neoadjuvant treatment in a very well-established subgroup of TNBC patients. Our results are in line with most of the studies published in the literature, supporting a benefit in pCR from the inclusion of platinum compounds to neoadjuvant regimens [30].

Since *BRCA*- mutated tumours represent up to 25% of all TNBC [19], it would be important to establish predictive factors that allow for patient selection for platinum- based treatment. *BRCA* status is being studied as a predictive biomarker of response to platinum agents, based on preclinical evidence that tumours harbouring *BRCA* deficiency are homologous recombination deficient and consequently, more sensitive to DNA damaging agents like platinum salts. Nevertheless, the predictive role of *BRCA* status is still uncertain in this setting. Recent evidence suggests favourable results in treating *BRCA* patients with platinum chemotherapy agents: the use of neodjuvant chemotherapy with cisplatin in *BRCA* 1 mutation TNBC patients accomplished a pCR rate of 61% [30]. Another recent study, which included neoadjuvant chemotherapy with anthracycline and taxane- containing regimens in TNBC women, has shown a pCR rate of 53.8%. Of the patients achieving pCR, 60.6% were *BRCA* negative and 39.4% *BRCA* positive [31]. This difference in pCR rates reveals the low efficacy of traditional chemotherapy regimens in this sub group of patients. Previous results of meta-analyses evaluating pCR in TNBC after platinum –based neoadjuvant chemotherapy have shown a beneficial effect [32, 33].

This meta-analysis is innovative as it compares the effect of platinum- based chemotherapy in TNBC patients with and without *BRCA* mutations. We found a trend towards higher efficacy of platinum-based regimens in mutated TNBC compared to non-mutated patients.

This result is in line with a recent randomized phase II trial, ADAPT TN, which has shown higher pCR in the neoadjuvant setting with the addition of carboplatin to a taxane based regimen in TNBC (45.9% vs 28.7%) [34], but that did not indicate the *BRCA* status. Other published results support a statistically significant benefit in TNBC pCR with the addition of a platinum agent to standard chemotherapy; [10, 11] however, once again the subpopulation of *BRCA* mutated patients was not discriminated in the studies.

On the other hand, the secondary analysis of GeparSixto trial showed that the response to carboplatin was independent from germline *BRCA* status [21]. When comparing pCR rates in the non-carboplatin arm the results were 66.7% vs 36.4% (OR = 3.50; 95% CI [1.39–8.84]; *p* = 0.008) for germline *BRCA* mutation patients and wild-type patients respectively. Surprisingly, the addition of platinum did not increase pCR rate in the germline *BRCA* group (65.4%); in contrast, carboplatin increased response rates in patients without *BRCA* mutations (55.0%), (OR = 1.55; 95% CI [0.64–3.74]; *p* = 0.33). In addition a recent meta-analysis [35] which included the GeparSixo study [21] and also the BrighTNess trial [28] compared platinum-based vs platinum- free neoadjuvant chemotherapy in TNBC patients. A total of 96 *BRCA*- mutated patients was included, but contrary to our results, the addition of carboplatin was not associated with a significant increase in pCR rate (OR = 1.17; 95% CI [0.51–2.67], *p* = 0.711). Unexpectedly, among patients without *BRCA* mutations there was an advantage in the use of platinum based chemotherapy (OR = 2.72, 95%CI [1.71–4.32], *p* < 0.001). Nonetheless, the authors stated that no conclusions could be drawn because of the small number of patients included. Furthermore, this study was designed to evaluate the effect of platinum-based versus platinum-free neoadjuvant chemotherapy in TNBC patients, and not the effect of platinum compounds in mutated vs non-mutated patients.

When searching for relevant abstracts in the conference proceedings from ESMO and ASCO, we have found reports of many ongoing studies on the subject. Fontaine et al. [36] in ESMO 2017 concluded that the addition of platinum to neoadjuvant treatments presented a pCR rate in the breast and axilla as high as 60% in early TNBC patients is obtained. Correlation with genomic homologous recombination deficiency HRD is ongoing.

A randomized phase III trial (PEARLY) [37] compared anthracyclines followed by taxane versus anthracyclines followed by taxane plus carboplatin as neoadjuvant therapies in patients with early triple-negative breast cancer. This study is expected to be published in 2023, with the patients being randomized on *BRCA* status.

At ESMO 2018 the results of the GeparOcto study [38] were presented, which evaluated the germline mutation status and therapy response in patients with TNBC. In this phase III study the patients with TNBC were randomized to receive treatment with intensified dose-dense epirubicin, paclitaxel, and cyclophosphamide, or weekly paclitaxel/liposomal doxorubicin, plus carboplatin. They concluded that *BRCA1/2* germline mutations represent a predictive biomarker for the achievement of pCR after neoadjuvant anthracycline-taxane-containing chemotherapy.

The PARTNER study [39] presented at ASCO 2017 which intended to evaluate the efficacy of the addition of olaparib to platinum-based neoadjuvant chemotherapy in triple negative breast cancer and/or germline mutated *BRCA*. Patients were randomised (1,1:1) to either control carboplatin and paclitaxel or one of two research arms with the same chemotherapy regimen but with two different schedules of olaparib.

Frovola et al. [40] presented at ASCO 2016, studied the role of different germline *BRCA* mutations in response to platinum-based chemotherapy in patients with triple-negative breast cancer. Early and locally advanced TNBC *BRCA* mutated cancers were treated with dose-dense combination of doxorubicin, cisplatin and paclitaxel followed by surgery and the study concluded that *BRCA* mutations may predispose different responses to platinum-based chemotherapy.

All of these recent preliminary and unpublished studies attest the relevance of the topic and the continuum debate on the field.

There are some limitations in our review. First, there was wide variation in the chemotherapy regimens regarding number of cycles, frequency, and agents used other than platinum compounds. Different types of treatments were included, such as eribulin, a non-taxan microtubule dynamics inhibitor, [41] or iniparib, a PARP 1 inhibitor [42].

Another limitation concerns to the small number of RCTs that have been included, only two studies [21, 28], which however comprise an evaluation of a significant number or patients. Furthermore, as we have selected only subgrups of the overall samples we have obtained homogeneity.

We must emphasize that our study only included patients with germinal mutation on *BRCA*1/2. Other putative “BRCAness” subgroups such as basal phenotypes, homologous recombination (HR) deficiency, *BRCA1* methylation or low levels of *BRCA1* mRNA were not assessed. However, a recent phase III trial in advanced TNBC showed a positive effect of carboplatin on germline *BRCA1* mutation patients but not on the *BRCA1* methylation, *BRCA*1 mRNA –low levels or HR deficiency subgroups [43]. Moreover, in the total population of TNBC patients, no evidence of a superior response to carboplatin was observed, when compared with the standard of care. In contrast, the subgroup of patients with germline *BRCA* 1 mutation showed a clear benefit of carboplatin, which doubled the effect of docetaxel [43].

The main strength of our review was the choice of a specific population of TNBC, resulting in a small publication bias and low heterogeneity of the sample, confirmed both by the value of the *I*^*2*^ heterogeneity statistics and the funnel-plot.

Despite the differences between studies regarding chemotherapy drugs, dose, number of cycles, patients’ age, tumour stage and study design, our results highlight the benefit of platinum in a selective subgroup of TNBC.

It is important to point out that TNBC constitutes a heterogeneous group of diseases so future studies are required in order to further individualize treatment.

## Conclusions

This meta-analysis shows that the addition of platinum to chemotherapy regimens in the neoadjuvant setting tend to increase pCR rate in *BRCA-*mutated as compared to wild-type TNBC patients (OR = 1.459 CI 95% [0.953–2.34] *p* = 0.082). The OR obtained indicates a clinical benefit on the addition of platinum compounds in *BRCA* mutated TNBC patients, but due to the small number of patients was not possible to achieve statistical significance and more studies are needed to definitely confirm the utility of platinum compounds in this setting.

## Abbreviations

CI: Confidence interval
OR: Odds ratio
pCR: Pathologic complete response
TNBC: Triple negative breast cancer
